# Thrombomodulin as a Physiological Modulator of Intravascular Injury

**DOI:** 10.3389/fimmu.2020.575890

**Published:** 2020-09-16

**Authors:** Kanako Watanabe-Kusunoki, Daigo Nakazawa, Akihiro Ishizu, Tatsuya Atsumi

**Affiliations:** ^1^Department of Rheumatology, Endocrinology and Nephrology, Faculty of Medicine and Graduate School of Medicine, Hokkaido University, Sapporo, Japan; ^2^Faculty of Health Sciences, Hokkaido University, Sapporo, Japan

**Keywords:** thrombomodulin, damage-associated molecular patterns, disseminated Intravascular coagulation, neutrophil extracellular traps, high mobility group box 1, immunothrombosis

## Abstract

Thrombomodulin (TM), which is predominantly expressed on the endothelium, plays an important role in maintaining vascular homeostasis by regulating the coagulation system. Intravascular injury and inflammation are complicated physiological processes that are induced by injured endothelium-mediated pro-coagulant signaling, necrotic endothelial- and blood cell-derived damage-associated molecular patterns (DAMPs), and DAMP-mediated inflammation. During the hypercoagulable state after endothelial injury, TM is released into the intravascular space by proteolytic cleavage of the endothelium component. Recombinant TM (rTM) is clinically applied to patients with disseminated intravascular coagulation, resulting in protection from tissue injury. Recent studies have revealed that rTM functions as an inflammatory regulator beyond hemostasis through various molecular mechanisms. More specifically, rTM neutralizes DAMPs, including histones and high mobility group box 1 (HMGB1), suppresses excessive activation of the complement system, physiologically protects the endothelium, and influences both innate and acquired immunity. Neutrophil extracellular traps (NETs) promote immunothrombosis by orchestrating platelets to enclose infectious invaders as part of the innate immune system, but excessive immunothrombosis can cause intravascular injury. However, rTM can directly and indirectly regulate NET formation. Furthermore, rTM interacts with mediators of acquired immunity to resolve vascular inflammation. So far, rTM has shown good efficacy in suppressing inflammation in various experimental models, including thrombotic microangiopathy, sterile inflammatory disorders, autoimmune diseases, and sepsis. Thus, rTM has the potential to become a novel tool to regulate intravascular injury via pleiotropic effects.

## Introduction

Endothelial cells coordinate vascular homeostasis, including vessel permeability, provision of a lining surface, and coagulation system regulation. To prevent unnecessary clotting, the endothelium expresses anti-coagulant factors, such as tissue factor pathway inhibitor and thrombomodulin (TM), and regulators of platelet activation, such as nitric oxide, prostacyclin, and ADPase, at steady state. When traumatic vascular injury occurs, platelet aggregation and the activated blood coagulation system invoke a thrombus to prevent blood loss. Moreover, damaged endothelium reduces the expression of anti-coagulant and platelet molecules, and releases pro-coagulant factors via the activation of nuclear factor-kappa B (NF-κB) signaling, consequently enhancing thrombus formation. Meanwhile, during non-traumatic intravascular injury, including disseminated intravascular coagulation (DIC), atherosclerosis, and thrombotic microangiopathy (1), the endothelium collaborates with the blood coagulation system and platelets to cope with the traumatic situation, possibly forming an unwanted thrombus. In addition, cross-talk between the activated coagulation system and inflammatory signaling leads to mutual amplification (2). Accordingly, damage-associated molecular patterns (DAMPs) released from injured tissues and blood cells activate the innate immune system and elicit vascular inflammation (3, 4). DAMPs directly activate platelets and indirectly induce platelet aggregation via interaction with neutrophils, leading to an enhancement of the pre-existing pro-coagulant state. This series of events of coagulation and blood cell activation, collectively referred to as immunothrombosis, is supposed to physiologically enclose and effectively kill invading microbes as part of an innate immune response (5). The structural basis of the immunothrombotic clot is formed by fibrin, consisting of coagulant factors, platelets, and leukocytes. The immunothrombus can also be involved in the development of non-infectious diseases, including ischemia-reperfusion, drug-induced tissue damage, autoimmune diseases, and cancer as an executor of intravascular injury. In the pro-coagulant state, TM derived from altered endothelium serves to maintain vascular homeostasis by participating in the coagulation system. Furthermore, TM possesses multiple regulatory properties against inflammation beyond its anti-coagulant effect, which could possibly contribute to the termination of intravascular injury (6, 7).

## Anti-Coagulant Effects of TM in Vascular Biology

TM is a transmembrane glycoprotein encoded by the *THBD* gene, and it is expressed on endothelium, immune cells (including neutrophils, macrophages, monocytes, and dendritic cells), vascular smooth muscle cells, keratinocytes, and lung alveolar epithelial cells (8–10). The structure of TM comprises five domains; each domain possesses a different function. Surface domains are a lectin-like domain (TMD1), a domain with six epidermal growth factor-like structures (TMD2), and a serine- and threonine-rich domain (TMD3). Certain stimuli, including tissue factor, orchestrate the coagulation cascade and produce thrombin as a coagulant executor. In response to thrombin production, thrombomodulin on the endothelium acts as a thrombin receptor to reduce the ability of thrombin that converts fibrinogen to fibrin and activates platelet. The thrombin-thrombomodulin complexes activate protein C and the activated protein C (APC) inactivates Va and VIIIa, resulting in the suppression of thrombin generation (11, 12). As such, TM naturally serves to terminate excessive intravascular coagulation.

## Anti-Inflammatory Effects of TM

The surface TMD1 domain has no anti-coagulant effects, but has various anti-inflammatory properties. TM directly acts as a natural regulator of inflammation via its lectin-like domain TMD1 by (1) inhibiting leukocyte-mediated intravascular injury, (2) neutralizing DAMPs, including high mobility group box 1 (HMGB1) protein and histones, (3) binding to bacteria-derived components, and (4) suppressing the complement system. (1) Transgenic mice with a genetically deleted TMD1 domain showed increased mortality in endotoxin-induced sepsis, together with the finding that adhesion molecule expression and neutrophil infiltration were increased in TMD1-deficient endothelium (13). *Ex vivo* studies have shown that additional TMD1 binds to endothelial antigen during inflammation, competitively inhibiting leukocyte migration and adhesion (14). Furthermore, we (15) showed that recombinant TM (rTM), containing TMD123, directly binds to neutrophils via the macrophage-1 antigen (Mac-1) receptor, and thus inhibits neutrophil activation. In addition, rTM affects lymphocytes to inhibit pro-inflammatory cytokine/chemokine production during an inflammatory response. (2) Necrotic parenchymal cells and neutrophil extracellular traps (NETs) release HMGB1 and histones into the extracellular space. The former is a nuclear chromatin-binding protein that transduces intracellular pro-inflammatory signals via toll-like receptor 4 (TLR4) and the receptor for advanced glycation endproducts (RAGE) (16). The latter exerts distinct biological effects, including direct cell toxicity, exacerbation of immune responses via TLR stimulation, and the activation of platelets, consequently exacerbating DIC, thrombosis, post-ischemic organ damage, and sepsis (17, 18). TM potentially neutralizes these DAMPs, attenuating intravascular injury and organ damage (19, 20). (3) The TMD1 domain potentially binds to the Lewis Y antigen of lipopolysaccharide (LPS) that has pro-inflammatory properties, as it can interact with CD14 and TLRs, thus inhibiting excessive inflammatory responses (21). (4) TM and its TMD1 domain regulate the complement system by eliciting complement-inhibitory signals (22). Abnormal complement activation leads to endothelial dysfunction, including thrombotic microangiopathy. TM may negatively regulate the alternative complement pathway by enhancing complement factor I-mediated inactivation of C3b. In addition, TM interferes with thrombin-mediated complement factor C5 activation, which involves the production of anaphylatoxin, and the formation of a membrane attack complex. TMD2 and TMD3 also exert indirect anti-inflammatory effects via APC production, which activates protease-activated receptor-1 on the endothelium to induce cell protection by inhibiting NF-κB signaling (23). Furthermore, TM-thrombin binding enhances the activation of thrombin activation of fibrinolysis inhibitor (TAFI) that degrades bradykinin and complement factors (24), contributing to the regulation of inflammation. Collectively, TM regulates inflammation, the complement system, and endothelial protection in addition to anti-coagulation during intravascular injury, consequently preserving intravascular homeostasis.

## NETs and TM

**Figure 1:**
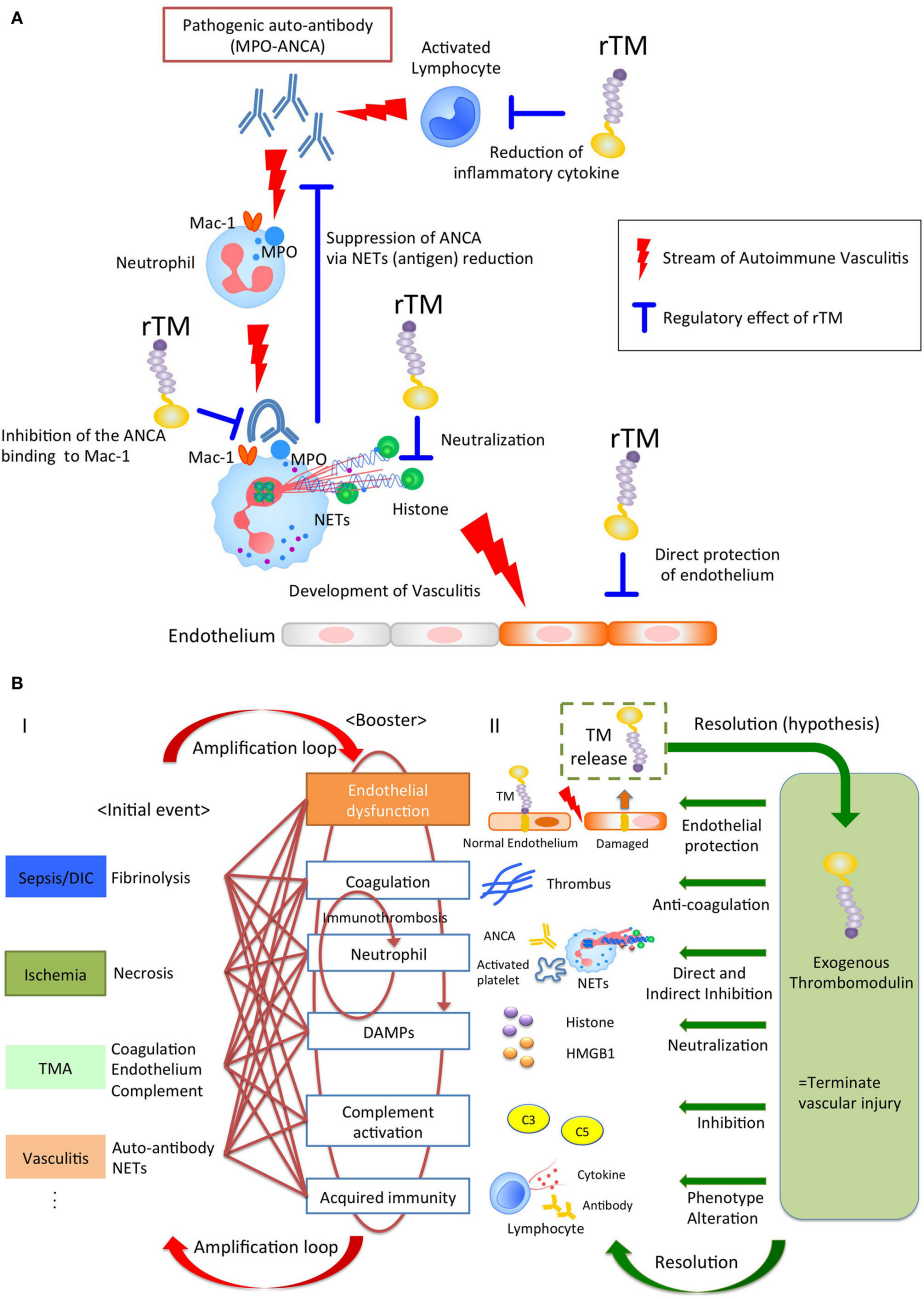
**(A)** The pleiotropic effects of rTM in autoimmune vasculitis. Pathogenic anti-neutrophil cytoplasmic antibody (ANCA) produced by lymphocytes binds to neutrophil antigen, inducing neutrophil extracellular traps (NETs). The NETs components cause vasculitis and could become auto-antigens, resulting in the further ANCA production. rTM suppresses the pro-inflammatory lymphocytes and inhibits the ANCA binding to Mac-1 on neutrophil, resulting in the suppression of NETs, which leads to the reduction of auto-antigens and ANCA production. Furthermore, rTM neutralizes cytotoxic extracellular histones in NETs and directly protects endothelium. Collectively, rTM could regulate the multiple points in pathogenesis of autoimmune vasculitis. **(B)** Thrombomodulin terminates auto-amplification of intravascular injury. (I) Intravascular injury in sepsis, ischemia-reperfusion injury, thrombotic microangiopathy, and vasculitis develops due to fibrinolysis, necrosis, coagulation/endothelial dysfunction, and neutrophil activation, respectively, as an initial event. In the next step, these events appear jointly with endothelial dysfunction, coagulation, neutrophil activation, damage-associated molecular patterns, complement activation, and acquired immunity to exacerbate the disease. In particular, immunity and coagulant systems collaborate to generate robust immune-thrombi, which accelerate intravascular injury, leading to an amplification loop. (II) Thrombomodulin is released into the intravascular space after endothelial injury and serves to counteract excessive coagulation and inflammation via its pleiotropic effects.

Various stimuli induce NETs through their own NETs-signaling mechanisms. However, regardless of the type of trigger, the NETs resulting from it could become major sources of DAMPs, and act as initiators of immunothrombosis in the face of intravascular injury (25, 26). Thus, NETs have the potential to become a therapeutic target for treatment of immunothrombosis-related diseases. Previously, rTM has been reported to downregulate several types of NET formation. Shimomura et al. showed that rTM inhibited NET formation following treatment with LPS-primed platelets by suppressing TLR4 signaling (27, 28). Studies by Shrestha et al. (29) indicated that rTM treatment ameliorated histone-induced sepsis by neutralizing extracellular histones and suppressing the formation of NETs (20). These previous reports implied indirect effects against neutrophils. Recently, we (15) could show the direct effect of rTM binding to neutrophils, which inhibited auto-antibody-mediated NET formation. In anti-neutrophil cytoplasmic antibody (ANCA)-associated vasculitis, pathogenic myeloperoxidase (MPO)-ANCA binds to MPO expressed on tumor necrosis factor α-primed neutrophils, and the Fc region of ANCA crosslinks with the Fcγ receptor coupled with Mac-1 on neutrophils to activate spleen tyrosine kinase signaling and ROS production, which results in peptidylarginine deiminase 4 activation and NET formation (30–32). In this scenario, rTM binds to Mac-1 to competitively interfere with ANCA binding on neutrophils, and inhibits downstream signaling, which suppresses ANCA-induced NET formation. Thus, TM potentially has direct and indirect inhibitory effects on NET formation, which contributes to the resolution of intravascular inflammation and immunothrombosis (Figure 1A).

## Experimental Evidence of rTM-Mediated Resolution of Inflammatory Intravascular Injury [Sepsis, Ischemic Reperfusion Injury, Thrombotic Microangiopathy (TMA), and Macroangiopathy]

Of note, rTM containing all the extracellular domains acts not only as an anti-coagulant, but also displays anti-inflammatory properties, hence contributing to the resolution of various diseases (Figure 1B and Table 1A).

**Table 1A:** Experimental evidence on recombinant thrombomodulin (rTM, including TMD1, TMD23, and TMD123 domains) in animal disease models.

**Animal model**	**Outcomes**	**Mechanisms**	**References**
Histone-induced thrombosis (mouse)	Improved mortality and thrombosis	Neutralization of histones	(20)
Cecal ligation and puncture-induced peritonitis (rat)	Improved coagulopathy	Regulation of NETs	(33)
LPS-induced sepsis (mouse)	Improved mortality	Neutralization of HMGB1	(34)
Renal ischemia-reperfusion injury (mouse)	Improved lung injury (remote organ)	Regulation of NETs	(35)
Renal ischemia-reperfusion injury (rat)	Improved renal function and histology	Reduction of leukocyte infiltration	(36)
Intestinal ischemia-reperfusion (mouse)	Increased survival and liver damage (remote organ)	Regulation of NETs	(37)
Myocardial ischemia (mouse)	Reduced myocardial damage	Suppression of leukocyte-endothelial interaction and TLR signaling	(13, 38)
Lung ischemia-reperfusion injury (mouse)	Suppressed protein leakage	Reduction of leukocyte infiltration	(39)
Cerebral ischemic injury (mouse)	Reduced infarct volume	Neutralization of HMGB1	(40)
Anti-glomerular basement membrane glomerulonephritis (rat)	Improved histology	Neutralization of HMGB1	(41)
Experimental autoimmune encephalomyelitis (mouse)	Improved clinical and pathological severity	Neutralization of HMGB1	(42)
ANCA-associated vasculitis (rat and mouse)	Improved renal and lung vasculitis	Suppression of NETs, acquired immunity	(15)
Hemolytic uremic syndrome (mouse)	Improved mortality and renal histology	Regulation of the complement system	(43)
Diabetic glomerulopathy (mouse)	Improved nephrosis	Inhibition of the complement system and inflammasome	(44, 45)
Arthritis (mouse)	Improved arthritis	Complement inhibition	(46)
Acute respiratory distress syndrome (mouse)	Increased survival rate	Neutralization of HMGB1 and increase in regulatory T cells	(47)
Bleomycin-induced pulmonary fibrosis (mouse)	Improved lung damage	Inhibition of transforming growth factor-β1 and HMGB1	(48, 49)
Bronchial asthma (rat)	Improved lung function	Modulation of dendritic cells	(9)
Pre-eclampsia (rat)	Improved maternal and fetal conditions	Improvement of hypo-perfusion	(50)
Recurrent spontaneous miscarriage (mouse)	Improved fetal resorption	Increase of VEGF expression	(51)
Lung metastasis (mouse)	Inhibited invasion and metastasis of cancer cells	Thrombin-independent mechanism	(52)
Pancreatic cancer (mouse)	Suppressed tumor growth	Inhibition of NF-κB activation	(53)
Atherosclerosis (mouse)	Improved atherosclerotic change	Anti-autophagic action and inhibition of thrombin-induced endothelial activation	(54, 55)
Aortic aneurysm (mouse)	Suppressed aneurysm	Inhibition of HMGB1-RAGE signaling	(56, 57)

*ANCA, anti-neutrophil cytoplasmic antibody; HMGB1, high mobility group box 1; LPS, lipopolysaccharide; NETs, neutrophil extracellular traps; NF-κB, nuclear factor-kappa B; RAGE, receptor for advanced glycation end product; TLR, Toll-like receptor; VEGF, vascular endothelial growth factor*.

### Sepsis

Sepsis involves multi-organ dysfunction with systemic inflammatory processes, immune dysregulation, coagulopathy, and other physiological responses. Among these processes, NETs and necrotic cell-derived DAMPs directly injure the endothelium and contribute to the development of immunothrombosis through the activation of platelets, coagulation systems, and recruitment of neutrophils (17, 25, 89, 90). In a mouse histone-induced septic model, pretreatment with rTM reduced mortality rates by neutralizing histones (20). In a rat sepsis/peritonitis model (33) and a murine LPS-induced septic model (34), rTM controlled sepsis-related immunothrombosis by limiting abnormal hemostasis and NET formation.

### Ischemia-Reperfusion Injury (IRI)

IRI occurs in response to the physiological processes that accompany tissue ischemia with inadequate oxygen supply. This is followed by reperfusion that drives regulated necrosis and subsequent inflammatory responses, leading not only to local organ damage, but also to remote organ injury in the form of necroinflammation (91, 92). In the animal brain, heart, lung, and liver, rTM (the entire ectodomain with lectin-like domain TMD1) ameliorated IRI tissue damage via anti-inflammatory effects, including neutralization of HMGB1 and histones, subsequently triggering the TLR4 signaling pathway (13, 38–40, 93). In a mouse model of renal IRI, ischemia-initiated tubular epithelial cell necrosis released extracellular histones and induced NET formation, which further contributed to remote lung injury (94). Interestingly, rTM (35) and a histone-neutralizing antibody (94) ameliorated remote organ damage, but did not have sufficient effects on local kidney injury. Conversely, inhibition of regulated necrosis, including necroptosis, mitochondrial necrosis, and ferroptosis, rescued local kidney injury at primary lesions, but had less effect on remote organ injury compared with histone neutralization (94). The discrepancy between local and remote injury was compatible with the phenomenon observed in an rTM-treated intestinal IRI mouse model, in which rTM improved remote liver injury, but not local intestinal damage (37). These findings imply that primary necrotic organ injury might develop based on the intracellular signaling cascades arising in response to IRI, but remote organ injury might mainly be caused by DAMPs and inflammatory responses, which could provide a better understanding of DAMP-related IRI pathogenicity.

### TMA

TMA is characterized by thrombocytopenia, microangiopathic hemolytic anemia, and organ injury. The underlying pathogenesis of TMA is understood to be endothelial dysfunction, which is caused by bacterial toxins, deficiency or dysfunction of the complement system, deficiency or inhibition of ADAM-TS13, drug-induced reactions, and transplant complications (95). The major disorders are hemolytic uremic syndrome (HUS) and thrombotic thrombocytopenic purpura (TTP). *Escherichia coli* (O157:H7) induces HUS by producing Shiga toxins, which bind to endothelial cells in the kidney and brain, triggering them to undergo cell death by inhibiting protein synthesis and inducing the secretion of Von Willebrand factor multimers, which leads to endothelial injury and microthrombi (96, 97). In mice, TM deficiency (more specifically, lectin-like domain TMD1) exacerbated Shiga toxin-producing *E. coli* (STEC)-HUS (98). Furthermore, in STEC-HUS-induced mice, rTM treatment protected them from kidney injury by regulating intravascular inflammation, complement dysfunction, and the coagulation system (43).

### Macroangiopathy, Including Aortic Aneurysm

Aortic aneurysm develops in association with certain risk factors, including age, genetic predisposition, atherosclerosis, and smoking. The underlying pathogenesis is characterized by chronic vascular inflammation and degradation of collagen-producing structural matrix proteins, which weaken the aortic wall (99). In a CaCl_2_-induced abdominal aortic aneurysm model, rTM [entire ectodomain (56) and lectin-like domain TMD1 (57)] treatment ameliorated abdominal aortic aneurysm by suppressing inflammatory mediators, macrophage recruitment, and HMGB1-RAGE signaling. In an apolipoprotein E-deficient atherosclerosis model, rTM (TMD23) inhibited autophagy-related cell death of aortic endothelial cells, preventing the progression of atherosclerosis (54). *In vitro* studies have shown that rTM directly binds to fibroblast growth factor receptor 1 on the endothelium, which activates the phosphatidylinositol 3-kinase-AKT/mammalian target of rapamycin complex 1 signaling pathway, and inhibits autophagy (54, 100). These findings indicate that TM could potentially mediate large vessel homeostasis by controlling immunological responses and endothelium protection.

## Experimental Evidence of RTM as an Immune Modulator Beyond an Inflammatory Regulator

In previous sections, the anti-inflammatory effects of rTM against intravascular injury were mainly described. In autoimmune diseases, including Goodpasture's syndrome (41) and autoimmune encephalomyelitis (42), rTM ameliorated the disease by suppressing inflammation and neutralizing DAMPs. Interestingly, recent reports have indicated that rTM acts as an immune modulator in addition to serving as an inflammatory regulator. In our study, rTM affected acquired immunity as well as neutrophil activation to resolve autoimmune vasculitis (15). Pathogenic ANCA auto-antibodies play a pivotal role in the development of ANCA-associated vasculitis. In this regard, rTM binds to antibody-producing lymphocytes to alter their activities from pro-inflammatory to anti-inflammatory, which contributes to the reduction of ANCA production and the resolution of the disease. Furthermore, Takagi et al. (9) reported that rTM ameliorated the ovalbumin-induced asthma model by regulating pathogenic dendritic cells. In a graft-vs.-host disease (GVHD) model, rTM increased regulatory T cells via the induction of anti-apoptotic Mcl-1 expression, resulting in the improvement of GVHD (101, 102). Similarly, rTM ameliorated acute respiratory distress syndrome in mice with an increase in regulatory T cells (47). Van De Wouwer et al. (46) showed that rTM (lectin-like domain TMD1) improved mouse arthritis by suppressing excessive inflammatory responses by macrophages and complement activation. As such, rTM could potentially modulate systemic acquired immunity in response to intravascular injury separately from maintaining local vessel homeostasis.

## Clinical Evidence for rTM-Based Strategies

Several studies have reported the serum TM level to examine its role in various diseases. Sepsis (58), ischemic disease (63), and autoimmune diseases (64) showed high levels of soluble TM in serum and plasma that reflected prevailing endothelial injury, indicating that soluble TM levels might be useful for disease diagnosis (Table 1B). Does endogenous soluble TM protect from intravascular injury in human disease? In coronary heart disease, the level of soluble TM is inversely correlated with disease severity (77), implying that endogenous TM might contribute to the resolution of this disease. However, because soluble TM is released from damaged endothelium to counteract the disease, soluble TM levels are often found to increase with disease severity (Table 1B) (77). Meanwhile, genetic polymorphisms of TM could influence the disease beyond the quantity of TM, which might explain the discrepancy between the titer and disease (103). It might be difficult to determine the role of endogenous TM based on soluble TM levels. However, the efficacy of additional TM has been clinically revealed with regard to several diseases during the past two decades.

**Table 1B:** The levels of serum thrombomodulin (TM) in diseases with intravascular injury.

**Disease**	**References**	**Levels of sTM**	**Correlation**	**With**
Sepsis/DIC	(58)	–	Positive	DIC, multiorgan dysfunction, mortality
	(59)	Increase	Positive	Disease severity, mortality
Cerebral infarction	(60)	Increase	–	–
	(61)	No change	Inverse	Disease severity
	(62)	Increase	No	Disease severity
	(63)	Increase	Positive	Disease progression
<Autoimmune disease>				
Systemic lupus erythematosus	(64–66)	Increase	Positive	Disease activity
ANCA-associated vasculitis (GPA)	(67, 68)	Increase	Positive	Disease activity
ANCA-associated vasculitis (GPA or MPA)	(69)	–	Positive	Disease activity
ANCA-associated vasculitis (EGPA)	(70)	–	Positive	Disease activity
Diabetes	(71)	Increase	Positive	Nephropathy and/or Retinopathy
	(72–74)	Increase	Positive	Nephropathy
	(75)	–	Inverse	Risk of type 2 Diabetes
<Cardiovascular disease>				
Coronary heart disease	(76)	No change	–	–
	(77, 78)	–	Inverse	Risk of coronary heart disease
	(79)	Increase	–	–
	(80)	–	Positive	Risk of coronary heart disease
	(81)	–	None	Risk of coronary heart disease
Atherosclerosis	(82, 83)	Increase	–	–
	(77)	–	Positive	Risk of carotid atherosclerosis
	(84)	Increase	Positive	Sclerotic changes in hypertensive retinopathy
	(85)	Increase	Positive	Intima-media thickness
Aortic aneurysm	(86)	Increase	Positive	Risk factors for atherosclerosis
Pre-eclampsia	(87, 88)	Increase	–	–

*ANCA, anti-neutrophil cytoplasmic antibody; DIC, disseminated intravascular coagulation; EGPA, eosinophilic granulomatosis with polyangiitis; GPA, granulomatosis with polyangiitis; MPA, microscopic polyangiitis; sTM, serum thrombomodulin*.

### DIC

In randomized, double-blind clinical trials, in which patients with DIC associated with hematologic malignancy or infection were treated with rTM or heparin, rTM improved DIC, and alleviated hemorrhagic complications compared with heparin (104). Although rTM therapy did not reduce all-cause mortality in a large clinical trial, *post-hoc* subgroup analysis stratified by the persistence of abnormal coagulation showed a tendency to decrease mortality (105). Meanwhile, a one-arm prospective trial revealed the effectiveness of rTM in solid tumor-associated DIC (106). Moreover, rTM administration could potentially be useful for treatment of obstetric DIC. During pregnancy, placental abruption, bleeding, and hypoxia could drive DIC underlying obstetric disorders, which is associated with maternal and fetal morbidity and mortality (107). A retrospective comparative study revealed that rTM significantly improved clinical and laboratory findings compared with controls in patients with obstetric DIC (108).

### TMA

TMA is associated with high mortality regardless of the underlying disease, including HUS, TTP, transplant complications, and drug side effects. In a case series of three patients with HUS, rTM ameliorated clinical outcomes with improvements reflected in reduced platelet counts and excessive complement activation (109). Furthermore, rTM could be beneficial for patients with transplant-associated (TA)-TMA. The latter is a severe complication after hematopoietic stem cell transplantation. The putative etiology is endothelial injury, which is caused by cytotoxic agents, infections, and GVHD (110). A case report (111) and retrospective cohort study (112, 113) showed the effectiveness of rTM with favorable clinical features and overall survival. Likewise, hepatic sinusoidal obstructive syndrome shows clinical manifestations characterized by hepatomegaly, jaundice, ascites, fluid retention, and thrombocytopenia following hematopoietic stem cell transplantation, with pathogenesis mechanisms similar to those of TA-TMA (114). Moreover, patients treated with rTM showed remission and survival rates equivalent to that of patients receiving defibrotide, which is the only recommended therapy for sinusoidal obstructive syndrome (115).

### Acute Exacerbation of Idiopathic Pulmonary Fibrosis (AE-IPF)

AE-IPF is a lethal condition associated with endothelial damage and abnormalities of the coagulation system (116, 117). HMGB1 is involved in the pathophysiology of pulmonary fibrosis (48). Furthermore, NETs are identified in the bronchi of patients diagnosed with AE-IPF, and are believed to contribute to disease progression (118). Kataoka et al. (119) reported that rTM therapy resulted in improved mortality rates compared with the control group (rTM vs. control: 30 vs. 65%). However, similar to the sepsis clinical trial, a large randomized phase III study in patients with AE-IPF did not show the superiority of rTM using the state of the control as primary endpoint (120). The cause is thought to be the heterogeneous pathology in the comparison group. Therefore, an appropriate study protocol with stratified risk factors is required.

### Clinical Perspectives of rTM Therapy via the Anti-inflammatory and Immune-Regulatory Effects

Although the efficacy of rTM has not been clinically shown in autoimmune disease and inflammatory disorder, several experimental data represent the potential to overcome these diseases. *In vitro* and animal studies indicate that rTM possesses the direct immunomodulatory effects in innate and acquired immunity independently of anti-coagulant effect (9, 15). Based on animal studies (Table 1A), rTM is being clinically expected to contribute to resolving diseases with inflammation including diabetes mellitus, arthritis, bronchial asthma, and ischemic-reperfusion injury. In particular, autoimmune ANCA vasculitis, which is characterized by immune dysregulation and intravascular injury, might be a candidate for rTM treatment. However, the dosage of rTM in many experimental situations (15, 33, 41) is 15–50 times of therapeutic dosage in patients with DIC and the effective concentration as an anti-inflammatory and immune-regulatory property remains unclear. Thus, in the future the indications of rTM therapy and the suitable dosage with no serious complications such as bleeding tendency should be carefully addressed.

## Conclusions

Immunothrombosis during intravascular injury leads to organ damage and further intravascular injury via cellular and molecular signaling, including excessive inflammation, coagulation, and cell activation. rTM regulates the immunothrombosis to terminate inflammation/coagulation, neutralize DAMPs, and affect immunity. The administration of rTM has the potential to become a novel therapeutic strategy for various diseases associated with immunothrombosis-mediated intravascular injury.

## Author Contributions

KW-K and DN conducted the literature research and reviewed all articles. AI and TA edited the article. All authors contributed to the article and approved the submitted version.

## Conflict of Interest

The authors declare that the research was conducted in the absence of any commercial or financial relationships that could be construed as a potential conflict of interest.

## Abbreviations

AE-IPF: Acute exacerbation of idiopathic pulmonary fibrosis
ANCA: Anti-neutrophil cytoplasmic antibody
APC: Activated protein C
DAMPs: Damage-associated molecular patterns
DIC: Disseminated intravascular coagulation
EGPA: Eosinophilic granulomatosis with polyangiitis
GPA: Granulomatosis with polyangiitis
GVHD: Graft-vs.-host disease
HMGB1: High mobility group box 1
HUS: Hemolytic uremic syndrome
IRI: Ischemia-reperfusion injury
LPS: Lipopolysaccharide
Mac-1: Macrophage-1 antigen
MPA: Microscopic polyangiitis
MPO: Myeloperoxidase
NETs: Neutrophil extracellular traps
NF-κB: Nuclear factor-kappa B
RAGE: Receptor for advanced glycation endproducts
rTM: Recombinant thrombomodulin
STEC: Shiga toxin-producing *Escherichia coli*
sTM: Serum thrombomodulin
TAFI: Thrombin activatable fibrinolysis inhibitor
TA-TMA: transplant-associated thrombotic microangiopathy
TLR: Toll-like receptor
TM: Thrombomodulin
TMA: Thrombotic microangiopathy
TTP: Thrombotic thrombocytopenic purpura
VEGF: Vascular endothelial growth factor.
